# Personality neuroscience and psychopathology: should we start with biology and look for neural-level factors?

**DOI:** 10.1017/pen.2020.5

**Published:** 2020-05-05

**Authors:** Neil McNaughton

**Affiliations:** Department of Psychology, Brain Health Research Centre, University of Otago, Dunedin, New Zealand

**Keywords:** Personality, Psychopathology, Neuroscience, Evolution, Explanation

## Abstract

“Personality is an abstraction used to explain consistency and coherency in an individual’s pattern of affects, cognitions, desires and behaviors [ABCDs]” (Revelle, 2007, p. 37). But personality research currently provides more a taxonomy of patterns than theories of fundamental causes. Psychiatric disorders can be viewed as involving extremes of personality but are diagnosed via symptom patterns not biological causes. Such surface-level taxonomic description is necessary for science, but consistent predictive explanation requires causal theory. Personality constructs, and especially their clinical extremes, should predict variation in ABCD patterns, with parsimony requiring the lowest effective causal level of explanation. But, even biologically inspired personality theories currently use an intuitive language-based approach for scale development that lacks biological anchors. I argue that teleonomic “purpose” explains the organisation and outputs of conserved brain emotion systems, where high activation is adaptive in specific situations but is otherwise maladaptive. Simple modulators of whole-system sensitivity evolved because the requisite adaptive level can vary across people and time. Sensitivity to a modulator is an abstract predictive personality factor that operates at the neural level but provides a causal explanation of both coherence and occasional apparent incoherence in ABCD variation. Neuromodulators impact all levels of the “personality hierarchy” from metatraits to aspects: stability appears altered by serotonergic drugs, neuroticism by ketamine and trait anxiety by simple anxiolytic drugs. Here, the tools of psychiatry transfer to personality research and imply both interaction between levels and oblique factor mappings to ABCD. On this view, much psychopathology reflects extremes of neural-level personality factors, and we can view much pharmacotherapy as temporarily altering personality. So, particularly for personality factors linked to basic emotions and their disorders, I think we should start with evolutionary biology and look directly at conserved neural-level modulators for our explanatory personality constructs and only invoke higher order, emergent, explanations when neural-level explanation fails.

## 1. Background

### 1.1 Personality *neuroscience*: a problem

If (as a neuroscientist) I am to analyse personality, I must first ask those who study it “What, at the psychological level, are the things I must explain”. As in other areas of biology, this request is for a consistent surface-level condensation of a vast mass of data into rule-based patterns – a taxonomy. Without a coherent data summary, each microscopic datum must be analysed independently and controlled experiments are impossible. A good surface-level taxonomy allows a start. However, surface rules are not explanations of the causes that give rise to the observations nor to their variations in time and space. Critically, the organisation of observations via superficial taxonomies is not predictive.

The definition of personality that I have found most congenial^1^ comes from Revelle (2007, p. 37):

Personality is an abstraction used to explain consistency and coherency in an individual’s pattern of affects, cognitions, desires and behaviors [ABCDs]. … The task of the personality researcher is to identify the consistencies and differences within and between individuals … and … to explain them.

This makes clear that the things to be explained (for any kind of scientist) are patterns of ABCDs but also that “personality” for a scientist links to a set of inferred *explanatory* constructs. For a neuroscientist, the obvious place to look for personality (so defined) is deep in the brain and deep in evolutionary time (as I will explain). But, given the way levels of explanation can work, it could be that the proper location for “personality” (as a causal explanatory construct) should be at the psychological (likely “cognitive”) level. That is, personality may be genuinely emergent – in the same way as (GCAT) genetics is emergent at levels well above quantum mechanics and even above much biochemistry. Conversely, as I will argue is true *in the case of conserved emotional traits*, the proper location for the real “personality” (that theories attempt to account for) is at the neural level.

We start, then, with the idea that, for personality, there are sets of *consistent* and coherent *patterns* of ABCDs that are what we need to explain. The method used to derive coherence from a mass of (usually) questionnaire data has been factor analysis. This is an excellent magic that distils dimensions from data.

But (I remind you that I am a neuroscientist): Should our solution be *varimax* or *promax?* Early personality research tended towards varimax, but analysis of neuronal coherence makes more sense as promax. We can easily have distinct neural sources (each with its own primary cluster of highly correlated measures) that interact and so have some common variance that makes most sense in terms of a promax analysis (Young & McNaughton, 2009). That is, each source is represented by a distinct *and biologically independent factor*, but the surface effects of the factors in terms of measured behaviour or neural activity are oblique (and so partially related) not orthogonal (i.e. completely independent). Importantly, causal explanations may not map neatly to the surface patterns, and so neural explanations of traits may not map neatly to our normal trait language any more than neural explanations of cognition map neatly to our everyday words.

Particularly if we use oblique solutions, how should we anchor our factor dimensions? Given the surface-level picture presented by modern personality systems, should a *causal* analysis focus on a general factor of personality (Erdle & Rushton, 2010) or a metatrait or a domain or an aspect or a facet? As someone who started totally outside this area, I feel the capacity to even ask this question at the present time creates a major problem for the neuroscientist wanting to identify the things that neural dynamics should explain. There are those who would argue that this factor identification problem is solved by a general agreement as to factors and structure. But, ignoring the mathematics behind all this, is your preference for Big Five or HEXACO^2^ (Ludeke et al., 2019)? In this uncertain terrain, where can the neuroscientist start with a top-down *explanation* of personality?

What if we start with bottom-up biology instead? We can look for neural-level factors (in the sense of underlying causes of specific patterns of inter-personal surface variance) and then use these to anchor (and so potentially redefine the nature of) traits. Critically, the associated *state* neurobiology can predict trait variance in ABCDs. Likewise, we can easily link extremes of such variance to psychopathology (e.g., some types of “panic disorder” can reflect a trait of panic-proneness). ABCD variance can also be analysed using psychopharmacology’s therapeutic tools (with “anxiolytic” drugs that are not “panicolytic” marking out systems involved in some form of trait anxiety as opposed to forms of trait panic). Importantly, such neurobiology need not be linear (e.g., Burger & Lang, 2001), and so the predicted ABCD patterns may depart significantly from those captured by the fundamentally linear, surface-level, factor analysis particularly of lexically derived items (what I mean by the “lexical approach” is explained in the section on it below).

What I will say in the “argument” section that is the second half of this paper will be consistent in principle with the neural and evolutionary approaches taken by previous neuroscience-based theories of personality. So, I will review a selection of these theories in the next sub-section and finish with an overview of neural state systems (and implied trait control) that essentially combines the previous *state* approaches. I will also describe in the following subsection how generation of trait scales from these previous supposedly neural theories has nonetheless followed the conventional lexical approach with no biological anchoring. My final conclusion will be that this lexical derivation of scales is the opposite of what should be done with largely conserved emotion-related traits, however valid it may be for personality more generally.

### 1.2 Personality neuroscience: some existing theories

Previous neuroscience-based personality theories are rooted in analysis of emotion systems, which strongly links state and trait control. A first critical common assumption is that fundamental emotional systems are evolutionarily old and largely conserved, as is their long-term trait control and its linkage to neurological and psychiatric illness (Greene et al., 2020). So, from this starting point, it is reasonable to follow not only a Darwinian perspective on origins but also Darwin’s preference for using other animals’ emotional behaviour as a guide to human emotions since they are “less likely to deceive us”. A second critical common assumption is that emotions can be identified with specific brain circuits, although current personality-related theories differ both in their naming of emotions and traits and also, partly as a result of this differing terminology, in their neural mappings. A third critical common assumption (almost required by definition) is that we can view psychopathology, largely, as the result of extremes of emotion-related traits (and so it gives a guide as to the nature of those traits). That is, emotion systems are required because certain responses (e.g., blood clotting) are adaptive under some conditions (e.g., threat) but maladaptive at other times (e.g., tending to produce clots and so strokes). Excess or inappropriate activation of such a system will necessarily be dysfunctional. I will briefly review theories reflecting these common assumptions in the current section. However, the implications of evolutionary conservation for hierarchical system architectures and for neurohormonal control, and so personality, are not treated by these theories as fundamental and have not been used in the derivation of their related personality scale systems. So, I will spell out these implications in the rest of this paper.

A good starting point for those interested in neuroscience-based theories of personality is the recent book by Davis and Panksepp (2018, published after Panksepp’s death). This book provides an extensive review of prior biological theories, and its primary goal is to present the most recent version of Jaak Panksepp’s highly detailed biological theory of core systems underlying human personality (see also Burgdorf & Panksepp, 2006; Davis & Panksepp, 2018; Davis, Panksepp & Normansell, 2003; Montag & Panksepp, 2017; Panksepp, 1982, 1998, 2006).

I start with, and focus particularly on, Panksepp’s theory since it largely includes the key principles of many earlier theories and can be seen as dealing with neural foundations that are essentially common to other, more cortically oriented theories. Davis and Panksepp (2018) start from Darwin’s demonstration of

the value of comparative research highlighting the similarities of behaviors and emotions across species and in doing so moved us conceptually closer to finding answers about the source of human motivation and temperament. … In fact, at every level of analysis, scientists could expect to find observations that would generalize from one species of animal to another. (pp. 53–54)

They translate this to the neuroscience level as the view that

to truly understand the brain processes that all vertebrates share, one has to start at the bottom, focusing on ancient, warm-blooded, highly inquisitive, social and playful mammals such as rats … At the bottom of the brain, the functional circuitries and emotional similarities across species are much more dramatic than at the top of the brain. …[So] foundations of human personality will be better revealed through the study of the evolutionarily older subcortical emotional brain processes … Until we have a solid graph of the fundamental emotional brain systems we are born with, we are unlikely to be able to accurately understand how those basic systems are elaborated by language and culture. (pp. 99–102)

So, Panksepp started with not just subcortical but hypothalamic areas where “emotive-command systems are defined primarily by neural circuits from which *well-organized behavioral sequences can be elicited* by localized stimulation of brain tissue” (Panksepp, 1982, p. 412, my emphasis). These highly conserved lower level neural circuits have recently been termed “survival circuits” by LeDoux (2012). Panksepp used quite distinctive behaviours (flight, foraging, distress vocalisation and biting) to identify the nominal psychological nature of each system (respectively, fear, expectancy, panic and rage), and he used the location of the electrode tip to identify the neural substrate of the emotion system (respectively, anterior ventral hypothalamus, lateral hypothalamus, anterior basal hypothalamus and ventrolateral hypothalamus). The theory developed over time and

until now, seven primal emotions have been identified by Panksepp (1998; 2011 – largely using deep brain stimulation approach), which all could be of relevance to understand human personality. Among these are four emotional circuitries for positive emotions (SEEKING, LUST, CARE, PLAY) and three emotional circuitries for negative emotions (FEAR, RAGE/ANGER and SADNESS/PANIC). (Montag & Panksepp, 2017, p. 3).

Critically, such stimulations when occasionally applied to the same areas in “human brains yield comparable affective experiences” (Panksepp, 2011, p. 1).

Table 1 shows the key neural mappings for Panksepp’s systems. In adapting this table from its original (Panksepp, 2011), I have (a) grouped the basic emotional systems into positive and negative sets; (b) reduced the details of the key brain areas and (c) simplified the originally listed neuromodulators by, in particular, mostly omitting neurotransmitters that act locally on parts of a system while retaining neuromodulators and hormones that act more generally.

**Table 1.** Primary components of Panksepp’s theory of basic state emotional control systems, sensitivities in which could result in personality traits. Adapted and reduced in content from Figure 5 of Panksepp (2011). Affectively positive emotions are listed first

System	Key brain areas	Key neuromodulators
SEEKING/expectancy (general positive motivation)	Nucleus accumbens; VTA; lateral hypothalamus; PAG	Dopamine; opioids; neurotensin; orexin; etc.
LUST/sexuality	Cortico-medial amygdala; BNST; preoptic hypothalamus; VMH; PAG	Steroids; vasopressin; oxytocin; CCK
CARE/nurturance	Anterior cingulate; BNST; preoptic area; VTA; PAG	Oxytocin; prolactin; dopamine; opioids
PLAY/joy	Dorso-medial diencephalon; parafascicular area; PAG	Opioids; cannabinoids
RAGE/anger	Medial amygdala; BNST; medial hypothalamus; PAG	Substance P
FEAR/anxiety	Central and lateral amygdala; medial hypothalamus; dorsal PAG	CRF; CCK; etc.
PANIC/separation	Anterior cingulate; BNST; dorso-medial thalamus; PAG	Oxytocin; prolactin; opioids; CRF

BNST, bed nucleus of the stria terminalis; CRF, corticotropin releasing factor; VMH, ventromedial hypothalamus; VTA, ventral tegmental area.

One complication, in relation to neuromodulation, is that the SEEKING system includes in its key brain areas the primary dopaminergic centres (nucleus accumbens and ventral tegmental area), and these are included less because of some distinctive elicited behaviour and more for their capacity to support self-stimulation (i.e. the contingent production of any arbitrary conditionable behaviour). The other key brain areas listed are more processing nuclei than neuromodulatory systems. Panksepp also links the SEEKING system to dopamine as neuromodulator (and to positive emotion in general), with dopamine also being linked to CARE. However, likely because other monoamine systems do not produce the equivalent of self-stimulation or easily identifiable forms of emotional behaviour when stimulated, Panksepp did not originally identify them with specific emotions. While serotonin is important for fear, anxiety and depression, he links it to non-specific inhibition of behaviour and he links noradrenaline to non-specific excitatory behaviour (Panksepp, 1982).

This differs from the perspective of Robert Cloninger (Cloninger, 1987; Cloninger & Gilligan, 1987; Gardini, Cloninger & Venneri, 2009). Linking a longitudinal, clinical, standpoint with the neuropharmacology and neuroanatomy of conditioning, Cloninger identified three major brain systems that delivered “three dimensions of personality that were postulated to be genetically independent of one another” (Cloninger, Svrakic & Przybecky, 1993, p. 976). The three systems, mapped to a progressive phylogenetic increase in learning abilities, were (a) behavioural activation/novelty seeking; (b) behavioural inhibition/harm avoidance and (c) behavioural maintenance/reward dependence. Importantly, unlike Panksepp, he provided a simple mapping of his key systems to monoamine neuromodulators that provided system-specific overall control (respectively, dopamine, serotonin and noradrenaline). He saw these neuromodulators as both generating psychopathology and being the targets of therapeutic pharmacology.

Cloninger’s mapping of specific systems to control by specific neuromodulators makes the translation to personality, and particularly to its genetics, simpler than identifying specific systems with specific integrative brain structures. According to Gardini et al. (2009, p. 266),

personality dimensions, therefore, are said to be heritable, stable across time and there also is some indication that they might be modulated by normal variance in the level of functioning of the different neurotransmitter systems, especially by variations in the expression of the central monoamine systems. Individual differences in novelty seeking were found associated with variability in the level of activity of the dopaminergic system; individual differences in harm avoidance appeared linked to variability in the level of activity of the serotoninergic system; and individual differences in reward dependence seemed linked to variability in the level of activity of the noradrenergic system. Moreover, harm avoidance and reward dependence have been found to be associated with polymorphisms of serotonin and dopamine related genes such as the serotonin transporter linked promoter region (5-HTTLPR) and the dopamine transporter (DAT1) gene.

They also provided evidence for grey matter volume changes in a range of *cortical* areas that were linked to Cloninger’s original trait dimensions.

There is a third, distinct, type of approach to this area – initiated by Jeffrey Gray and with which I have long been associated – that focuses on brain nuclei in the same way as Panksepp but includes neuromodulators and higher level subcortical and cortical systems in a similar way to Cloninger. Panksepp himself has contrasted his views with ours in Davis and Panksepp (2018, pp. 234–237), saying:

Each of the 7 primary emotional-adaptation systems [see Table 1] … possess different conditioning parameters in the brain, with additional, distinct homeostatic affects … However, only the emotional affects are major contributors to personality development, even as they share many of the general brain chemistries for learning, such as glutamate and gamma-aminobutyric acid. … As each of these primary emotions are thought to have evolved for distinct survival issues, serving different purposes at different times in our emotional past, it is likely their learning parameters were evolutionarily adjusted to meet unique survival needs as well. … So, one of the more controversial positions taken by Gray and McNaughton (2000) was that fear and anxiety represented different brain systems and what we would call PANIC/Sadness was included with BIS anxiety. From our perspective, the natural emotional systems of the mammalian brain were not adequately integrated into the revision of the BAS and BIS. … Perhaps when one has worked with deep brain stimulation … it is easier to conceptualize the foundational emotional systems embedded in the subcortical brain than when starting from a learning theory perspective. Furthermore, why not study the behavioural, biological, and psychological mechanisms of each affective brain system separately, rather than lumping all “rewards” together into a single BAS and all “punishments” together into a single BIS [Behavioural Inhibition System] or FFFS [Fight, Flight, Freeze System]?

There are two key points to note in relation to this criticism: one psychological and one methodological. Taken together, they lead to the conclusion that Panksepp and Gray & McNaughton are largely talking about different things rather than disagreeing about the nature of the same things.

The first, psychological, point is that it is only when animals are close to contact with motivating objects (food, mates and predators) that distinct reinforcer-specific behaviours emerge. Importantly for the view that stimulation activates a motivational system rather than just eliciting behaviour, whether electrical stimulation produces a behaviour like attack depends on whether the test situation contains an appropriate object that then shapes the form of the attack. With the exception of the dopamine system and its contingent relation with self-stimulation, we can view the bulk of Panksepp’s results as dealing with proximity-specific behaviours and emotional states. It should also be noted that electrical stimulation will activate fibres of passage as well as control centres, which is particularly important for the interpretation of phenomena like self-stimulation. However, much motivated behaviour (such as lever pressing of a rat in an operant chamber) is quite general to different types of reinforcer (that generality was a main aim of Skinner in designing the operant chamber). Indeed, although they may normally appear fairly passive in sexual encounters, female rats will learn to press a lever to obtain a sexually potent male rat and lever pressing “latencies after ejaculation are longer than those after intromission, which in turn exceed those after mounts” (Bermant, 1961, p. 1771). Such reinforcer-general lever pressing will be controlled by much higher levels of the subcortex than the periaqueductal grey (PAG) (which is prominent in Table 1) and will involve cortical regions as well. Far from being unique and controversial, the fear (active avoidance; Fight, Flight, Freeze System (FFFS)) versus anxiety (passive avoidance; Behavioural Inhibition System (BIS)) distinction made by Gray and McNaughton is also made by Cloninger in the form of behavioural activation/novelty seeking versus behavioural inhibition/harm avoidance (Cloninger & Gilligan, 1987, Table 4, p. 468), although the factor labels, and the precise details of the proposed systems, are somewhat different between the theories. Although these two theories differ from Panksepp in emphasising neuromodulators and higher level structures, it should be noted that a neural fear/anxiety distinction can be made even within the PAG (see Figure 3 and Silva & McNaughton, 2019). Thus, the behaviours on which Panksepp focuses are linked to “survival circuits” that appear to be an important part of the bottom end of emotional control (Mobbs & LeDoux, 2018, see also the associated special issue) but do not appear to be the whole story since higher levels of subcortex and cortex are also important (LeDoux, 2012, 2015; but see also Panksepp, 2005). I would argue that emotional control involves hierarchical systems that can be viewed as essentially continuous between the PAG and the prefrontal cortex (Gray & McNaughton, 2000; McNaughton & Corr, 2004; Silva & McNaughton, 2019).

The second, methodological, point is that while the superstructure of Gray’s original theory owed much to learning (Gray, 1975), its foundation is in behavioural pharmacology (Gray, 1967, 1970, 1977). The psychology of the theory was couched in terms of reward and punishment when he translated it to personality (Gray, 1970; but see McNaughton & Corr, 2019 p. 129 for three quite distinct uses of the word “punishment” by Gray), which he treated as sensitivities in a conceptual nervous system that mirrors the central nervous system (Gray, 1972). Indeed, his personality translation excluded his fight/flight system (an intentional compound of RAGE and FEAR in Table 1), which at that time he saw as controlling only innate behaviour (Gray, 1971, see especially pp. 210–212). But, his key distinction between fear and anxiety, his postulation of the BIS and his identification of a likely neural substrate for the BIS (including prefrontal cortex and hippocampus) were all based on the actions of anxiolytic drugs. The crucial comparison in distinguishing a fear system from an anxiety system was the lack of effect of anxiolytics on escape and active avoidance, their impairment of passive avoidance and their *improvement* of two-way active avoidance (Gray, 1967, 1970, 1977). Even if we restrict ourselves to behaviours controlled by the more reinforcer-specific subcortical areas (and to elicitation by innate stimuli like predators), it is hard to see why Panksepp would want to allocate to a single homogenous system escape and active avoidance (elicited by an immediately present predator and sensitive to panicolytics but not anxiolytics) and risk assessment and defensive quiescence – elicited by a potential predator and sensitive to anxiolytics (Blanchard & Blanchard, 1990; Blanchard, Blanchard, Tom & Rodgers, 1990; Blanchard & Blanchard, 1990a; Blanchard, Griebel, Henrie & Blanchard, 1997; Griebel et al., 1995).

In what follows I will use a recently updated version of the Gray and McNaughton neurology (Silva & McNaughton, 2019) that, I would argue, essentially includes the core aspects of Panksepp’s and Cloninger’s ideas within its architecture (see Figure 4). Its lower levels owe much to data on electrical stimulation and overlap Panksepp’s ideas on fight, flight and freezing; it accommodates global control of emotional systems by monoamines consistent with Cloninger’s linkage of these to psychiatric disorders and it combines these points of view via the idea that emotional systems are hierarchically organised both in relation to psychological distance (Blanchard & Blanchard, 1990b) and neural caudal-rostral level (Graeff, 1994; McNaughton, 2011). For reasons given in the next section (and prompted by the comments of a referee), I will also offer a novel nomenclature for the key state brain systems involved and so for the traits that are the result of their long-term sensitivities to their inputs.

### 1.3 Personality neuroscience – the lexical approach?

The three types of theory that I have briefly surveyed above approach emotional systems and traits from different starting points (electrical stimulation, clinical description of genetically independent personality variants, and behavioural pharmacology mixed with learning theory). They also integrate elicited and learned behaviour in somewhat different ways. However, in my view, they differ more in the aspects of emotion control on which they focus (proximal elicitation, neuromodulation, and hierarchically organised modules) than they differ in details of their underlying neurobiology. All see the dopamine system, for example, as important for positive reinforcement, and the details of the lower levels of Gray and McNaughton’s FFFS/BIS overlap Panksepp’s RAGE/FEAR/PANIC systems. All see dysfunction of specific systems as underlying specific psychiatric disorders, and relatedly, all see a Darwinian evolutionary perspective as important and their systems as largely conserved (at least at the lower levels). However, none explicitly takes this evolutionary conservation as a starting point for any aspect of their state theories nor for the construction of their associated trait scales.

A key suggestion in what follows is that a closer look at the way neural systems will have evolved in relation to recurring adaptive challenges gives us good reason to see hormones and neuromodulators as key trait factors in general, with endogenous ligands of anxiolytic receptors as key factors for a form of trait anxiety in particular. Such an evolutionary perspective also, I believe, provides a basis for integrating the different biological theories. Importantly, all such theories are fundamentally linked to circuits in the real brain, and these circuits provide us a solid anchor for the higher level constructs employed by the theories and provide objective means of challenging them. What each theory says about a system must relate to its ground-level facts, and so appropriate objective data can allow only one (or none) of them to be right: there is no wiggle room. But, surprisingly, the theories reviewed above have not taken advantage of this. Like non-neural theories, they were all translated into personality research via a lexical approach. They have all failed to use the neural trait anchors that are fundamental to their state control details.

Here, and in what follows, I use “lexical approach” to refer to the development of scales of items that are created, and included, primarily based on their intended meaning as perceived by the experimenter. (In contrast, I will argue that we should anchor scales intended to assess theoretically defined biological systems directly to measures of the underlying biological construct of interest.) Factor analysis can ensure that experimenter-chosen items are linguistically related but, by itself, can do nothing more.

I do not take lexically derived scales to assess only language use. As has been emphasised to me by Colin DeYoung (email 13 February 2020):

I think we have to be very careful NOT to assume that if trait measures assess behavioral tendencies then these are merely “superficial patterns of verbal behavior”! Rather they are valid measures of behavioral tendencies more generally. I trust (because there is good validity evidence) that people who score high on extraversion do not merely *say* that they are more gregarious, assertive, talkative, dominant, excitable, enthusiastic, and joyful, but that they really ARE all of those things in their lives in general …. I hate the whole canard about the Big Five being “lexical”. That is totally irrelevant as long as they are measuring real, general patterns of behavior.

The requirement for validity in terms of behaviour that we could measure differently is an important caveat, but a number of measures achieve this. However, as he goes on to say,

let us not jump to believing that some questionnaire measures a neural sensitivity, or we are foregoing all of the crucial research that would actually allow us to understand the causes of personality! I will never accept that “FFFS sensitivity” and “BIS sensitivity” can be adequately measured by a questionnaire, and so I hate that Carver’s scale used those labels. Surely, we must have neural measures to determine FFFS and BIS sensitivity! [Neuroticism] is biological in the sense that it is caused by variation in biology. But again: something is not identical to its causes, and a behavioral tendency is not a neural sensitivity. … Personality questionnaires that ask people to describe themselves are of course descriptive, but my whole aim in science is to understand what causes the regularities in behavior that are described by those questionnaires.

As noted by a referee

“psycholexical” is commonly reserved for a purely inductive approach to questionnaire construction starting with a collection of all personality descriptive terms in a given language. [However], questionnaires like, for example, Carver and White’s (1994) BIS/BAS scales have been at least partly developed based on theoretical considerations.

I, like DeYoung, see such theoretically based scales (in particular, the ones we will consider in detail below) as nonetheless suffering terminally from using a lexical approach instead of anchoring items to neural measures validated for the systems of interest.

However, I think I differ from DeYoung when he says

I will never accept that “FFFS sensitivity” and “BIS sensitivity” can be adequately measured by a questionnaire. … From my perspective, you are conflating the traits with the parameters of mechanisms …. [FFFS] and BIS belong in the mechanism box (though Carver and others have tried to put them in the trait box), whereas [Neuroticism] belongs in the trait box.

Certainly, a direct neural measure of system sensitivity is preferable as a trait measure if we are talking about a defined neural system. However, I see no fundamental problem with a questionnaire scale where we choose the items by anchoring to a difficult or expensive to administer neural anchor, not the experimenter’s intuition. Conversely, I think that we will be able to see the neuroticism that Eysenck largely anchored to clinical patients, rather than linguistic assumptions, as fundamentally neural, once we know its causes. This may require an iterative approach to definition and scale development. The key negative point about the approaches considered below is that they take the same type of lexical approach to scale development as Carver in that they use no neural anchoring measures.

#### 1.3.1 Panksepp

Panksepp’s (Davis et al., 2003, p. 57, 59, 60) Affective Neuroscience Personality Scales,

modeled after the Spielberger State-Trait Personality Inventory (STPI), were constructed to estimate self-reported feedback concerning the putative influences of these six neurally based networks, which are labeled PLAY, SEEK, CARE, FEAR, ANGER, and SADNESS systems, along with a Spirituality scale and various filler questions. … PANIC has been modified to SADNESS to be more semantically straightforward. … Items for all scales were written with the goal of accessing personal feelings and behavior rather than more cognitive social judgments. For example, “I am known as one who keeps work fun” was preferred over “It is important to keep work fun”.

#### 1.3.2 Cloninger

For Cloninger’s 80-item self-report scale (Cloninger, 1987, p. 580; see also Cloninger, Przybeck & Svrakic, 1991)

questions were chosen to evaluate the behaviors that were thought to be characteristic of individuals deviant on one dimension … For example, two questions assessing the rapid, intuitive decision-making characteristic of novelty seekers are “I prefer to make decisions only after considerable thought” … and “I often act on hunches, momentary whims, or my intuition without making a detailed analysis of the facts”.

#### 1.3.3 Gray

Gray’s own Gray–Wilson Personality Questionnaire was largely lexically derived but, rather than tapping into cognitive/emotional content, was designed to assess human reactions to the equivalent of six key animal behaviour paradigms: approach, active avoidance, passive avoidance, extinction, fight and flight (Wilson, Barrett & Gray, 1989; Wilson, Gray & Barrett, 1990). Questions required yes/no answers but were not linked to simple behaviours and included “Are you inclined to curse audibly when things go wrong?”; “Do you visit the doctor for regular check-ups?”; “If a fight broke out in a bar where you were drinking, would you leave as fast as possible?”. However, rather than coalescing into his three key theoretical systems (which at that time were activation/approach + active avoidance, inhibition/passive avoidance + extinction and fight/flight = fight + flight) analysis extracted six factors and the conclusion that “the sources of individual variation that emerge as salient in human society may draw upon complex mixtures of biological systems” (Wilson et al., 1990, p. 1041). This mapping failure was likely to be at least in part due to problems with the assumed biology, which I corrected later with the 2000 update of the theory.

#### 1.3.4 BIS/BAS

A particularly popular set of scales (currently with over 6000 citations) based on Gray’s original (1982) theory is the BIS/BAS scales of Carver and White (1994). They started with a critique (based on lexical item content and factorial structure) of previous scales (including Cloninger’s Tridimensional Personality Questionnaire and the Gray–Wilson Personality Questionnaire, but not Panksepp’s Affective Neuroscience Personality Scales). Then, they created scales that they effectively validated against, for the BAS, *self-reported* happiness with an expected reward and, for the BIS, *self-reported* nervousness about an expected punishment. Thus, both their validation and their scale construction were lexical. For BAS, somewhat surprisingly, they saw sufficient lack of consensus as to how it would manifest (a lexical problem) to generate three distinct scales (drive, reward and fun seeking) for the supposed global sensitivity of one single system. For BIS, they tried to create items reflecting concern about negative outcomes (“I worry about making mistakes”) or reflecting sensitivity to them (“Criticism or scolding hurts me quite a bit”), but later analysis suggests that their resultant single “BIS” scale actually contains two (potentially true BIS- and true FFFS-related) factors (Heym, Ferguson & Lawrence, 2008; see also studies reviewed by Maack & Ebesutani, 2018). Maack and Ebesutani (2018), in a recent internet-based study, argue for the BIS/BAS containing both a single global BAS factor and a single global BIS factor. For BAS, “the majority of variance of individual BAS items was accounted for by a common, general BAS dimension” (p. 1 of 10); and for BIS, after correcting for method effects of reverse scored items, a single-factor solution appeared most parsimonious (but with the original two Heym sub-factors strongly *inversely* correlated at −0.96).

#### 1.3.5 Consensus?

I will not go into detail as to the problems of reconciling these (and the many other similar scales) that have been constructed based on supposed psychological aspects of what have to be in all cases the same fundamental real central nervous system. There is some variation in the neurology invoked by the three main theories, but there is much more variation in what scale constructors deem lexically appropriate as a measure even when restricted to one theory such as Gray’s (for critique see introductory parts of Carver & White, 1994). When comparing the theories at the lexical level, one has to battle with naming differences: FEAR does not, as one might expect, map to “Harm Avoidance”, which Cloninger himself saw as close to “behavioural inhibition”; PANIC can somehow become SADNESS so as to be “semantically straightforward”; and Davis and Panksepp see PANIC/SADNESS as having been included in BIS-controlled anxiety by Gray and McNaughton, who would see panic (as they define it) as opposite to, *and mutually inhibitory of*, anxiety (see also Graeff, 2011).

#### 1.3.6 We should avoid the lexical approach for neural theories

“Words are, of course, the most powerful drug used by mankind” (Kipling, 1923, cited by Canale, 2019), so it is not surprising that their use should generate a degree of confusion. Emotions as such, and emotion labels in particular, have always been hard to define, meaning different things to different people (McNaughton, 1989, 2018).

However, Darwin’s preference for studying animals in order to understand emotions was driven much more by the fact that, even when the words are simple, human verbal behaviour gives us a less reliable road to the mind than the non-verbal behaviour of humans or, preferably, other animals. For example, while autonomic awareness as reported via the Autonomic Perception Questionnaire correlates with “Manifest Anxiety” and with autonomic reactivity, it has *no relation* to the actual differences that can be observed in the capacity for autonomic perception between people (see Schandry, 1981).

This problem arises even with simple behavioural terms like approach and avoidance.

*Self-reported trait approach and trait avoidance are not consistently associated with behavior in experimental tasks* … leaving the question whether tasks and questionnaires indeed examine the same underlying systems unanswered. It should be mentioned that the Carver and White BIS/BAS-scales, which have been utilized in most of these papers, have been derived using lexical methods without behavioral validation, i.e. not showing a relationship to behavior but merely to self-report trait or state questionnaires (Carver & White, 1994). The BIS scale has further not been validated based on anxiolytic drugs, which had been initially used to fundamentally define the BIS as separate from the basic control of avoidance (Gray, 1977). (Fricke & Vogel, 2020, pp. 34, 36)

### 1.4 A new nomenclature for emotion systems

These various naming problems have driven me to offer, here, a new nomenclature for the neural systems of interest to personality theorists starting from a state motivation system base. In what follows, as a behaviourally oriented neuroscientist, I wanted to use as labels the simple (for me descriptive) terms approach, conflict and withdrawal. However, a referee pointed out that “it’s problematic to label ‘trait withdrawal’ as FFFS sensitivity given that within the [Big Five Aspect Scales] system, ‘trait withdrawal’ is clearly mapped to BIS – this is likely to confuse readers in personality psychology”. A similar problem arises in that when BAS is translated as Behavioural *Activation* System (rather than Behavioural *Approach* System), it is usually seen as including active avoidance (which would be linked to the FFFS). Likewise, a term such as “trait avoidance” leaves open the possibility of confusion of active avoidance (controlled by the FFFS) with passive avoidance (controlled by the BIS). In evolutionary terms, the *function* of “withdrawal” from threat can be achieved by active avoidance, escape, flight, fight and freezing (hence the usual naming of the system involved as FFFS); but I think that to talk about a flight/fight/freeze trait could confuse by both excluding avoidance and escape and conflating these higher level means of withdrawing from danger with lower level ones such as panic control. A final reason for a new nomenclature is that if the term BIS is retained for a *biologically defined* system then any new, neurally validated, scale could immediately be confused with the older, lexically derived, BIS scale of Carver and White (1994).

The new nomenclature (see Table 2) is derived as follows. The key characteristic of the BIS originally proposed by Gray (and as defined by anxiolytic drug action) appears to be the suppression of pre-potent goal-directed responding *when there is conflict between goals*. Here, a goal is seen as a combination of a situation with a motivation, and the goal can be either an attractor or a repulsor in respect to the way it elicits behaviour (Corr & McNaughton, 2012). Importantly, the anxiolytic-sensitive “behavioural inhibition” of the BIS does not include simple action stopping, which is insensitive to these drugs (McNaughton, Swart, Neo, Bates & Glue, 2013; Shadli, McIntosh, Glue & McNaughton, 2015; Shadli et al., 2019). Therefore, to avoid ambiguity, I propose a simple renaming of the system controlling *goal conflict* to the Goal Inhibition System (GIS) meaning a system that inhibits the effects on behaviour of both attractors and repulsors. This renaming has no substantive consequence for the original state neural architecture but allows a clear distinction for Reinforcement Sensitivity Theory (RST; see, Corr, 2008) between a neurally validated GIS scale and any part of the BIS/BAS scales (Carver & White, 1994) or similarly lexically constructed variants. Likewise, I propose that the fear-related system originally referred to as FFFS be termed the Goal Repulsion System (GRS); however, I retain FFFS as a label for a coherent set of survival circuits (LeDoux, 2012) at the bottom of the GRS hierarchy. Matching this, I propose renaming the BAS to the Goal Attraction System (GAS). This a) is a superficial change if “BAS” refers to neural structures and translates to “Behavioural Approach System”; b) is neurally somewhat different when translated to “Behavioural Activation System”; and c) clearly does not map directly to the existing *three* lexically derived BAS scales (Carver & White, 1994).

**Table 2.** Proposed new nomenclature for goal control systems

New name	The three major goal (situation + motivation) control systems			
	Goal **attraction** system	Goal **inhibition** system	Goal **repulsion** system	
Abbreviation	**GAS**	**GIS**	**GRS**	
Originally	**BAS**	**BIS**	**FFFS**	
Goal type	Attractor	Multiple conflicting	Repulsor	
Reinforcer type	Positive	Increased negative bias or decreased positive bias	Negative	
Distal behaviour	Attraction	Inhibition	Repulsion*	
Motivation type	Appetitive	Informative	Aversive	
*Survival function*	*Consumption*	*Conflict resolution*	*Defence*	
Proximal stimulus-specific behaviours**	Social aggression*** Eat (including Predation***)		Fight***	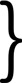 **FFFS**
		Defensive quiescence****	Freeze****	
	Drink Affiliate (e.g., groom) Mate (e.g., lordosis), *etc*.	Rear Risk assess, *etc*.	*Flight*	
			Disgust, *etc*.	

*Notes:*
Systems are fronto-limbic-hypothalamic and distinct from motor (action) control. GIS defined by anxiolytic drugs. Proximal GRS includes the basic fight/flight system as defined by Gray 1971/1978. GIS activated by upcoming *goal* conflict; GRS by negative *outcome* conflict. Survival circuits as in Ledoux (2012); see also Current Opinion in Behavioral Science 2018, 24, pp. 1–180.

* “Avoidance” (active = GRS/passive = GIS) and “withdrawal” are ambiguous.

** Trait control generally includes proximal-specific as well as distal-general behaviours.

*** “Fight” previously used ambiguously to include social aggression and predation.

**** “Freeze” previously used ambiguously to include defensive quiescence.

*Acknowledgment:* Thanks to Johnathan Williams, Colin DeYoung, and Philip Gable for discussion.

In the remainder of this paper, I will argue that (a) evolution will have generated some fundamental neural system control factors that are the causal basis of traits; (b) we should anchor our measures of such traits to biological measures of these factors; and (c) we should use these biologically defined factors to identify (and then, if necessary, rename) the patterns of ABCD for which they provide the explanation. This anchoring should avoid two problems with a lexically derived factor: (1) it may not map to its supposed biological system and (2) a narrowly defined “trait” may not map to any real ABCD trait at all (Cooper, 2019). The strong version of this argument applies only to largely conserved emotion-related traits. However, it can be argued that large-scale *connectional* systems are shared across all vertebrates. Importantly, “the high degree of crosstalk and association between these [connectional] systems at different levels supports the notion that cognition, emotion, and motivation cannot be separated – all of them involve a high degree of signal integration” (Pessoa, Medina, Hof & Desfilis, 2019, p. 296). Since the highest levels of cortical processing are also phylogenetically the oldest (i.e. agranular cortex), the conserved systems of interest may be cortical as well as subcortical and, potentially, deal with fundamentals of the highest levels of mental processing, with variation in isocortical areas simply adding filters equivalent to the addition of colour vision in primate lineages.

### 1.5 State versus trait biology

States and traits operate on very different timescales and have a number of features where they differ both in their phylogeny and in their current neural form. These state-trait differences are important for our ultimate conclusion: that trait neurobiology can be very simple while the associated state neurobiology can be very complex (particularly for moment-by-moment analysis).

A complex hierarchical system can deliver very complex patterns of ABCD (the result of transient states). However, if trait control is a matter of determining which level of a system will be in control for any given strength of input then the trait variation in an individual’s sensitivity to input may nonetheless have simple control. The picture I want to present is of trait control (sensitivity) applying to all levels of the system. In the case of the defence systems, one can view different neural levels as in control at different defensive distances (essentially a state-controlling variable) but where different individuals behave as if closer to or further from danger for any specific real distance depending on their trait sensitivity (higher or lower, respectively).

**Figure 1.**
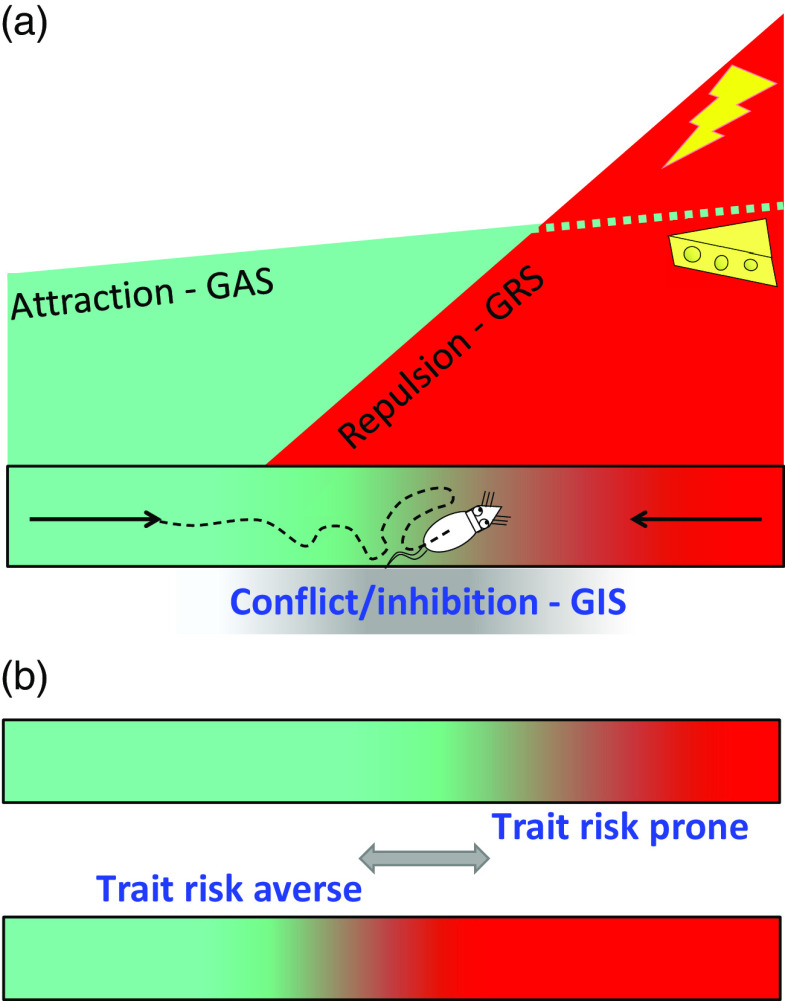
A: Attraction (green) and repulsion (red) have different motivational gradients (Brown, 1948; Kimble, 1961; Miller, 1944, 1959) dependent on distinct systems (GAS and GRS). As a result, a rat in an alley will start to run, attracted by its memory of food at the other end. However, as it becomes affected by repulsion, it will then slow down. When attraction and repulsion reach a position of balanced conflict between their goals, their associated behaviours are inhibited (grey) by a third system (GIS), and the rat will no longer move forward. Instead, it will dither or explore or engage in displacement activity such as grooming (figure adapted from McNaughton et al., 2016 with permission). B: Different rats have different levels of trait goal attraction (GAS sensitivity), trait goal repulsion (GRS sensitivity), and trait risk aversion (GIS sensitivity). So the highly dynamic observed behaviour varies systematically with the balance between these factors. For any fixed levels of attractor and repulsor activation (i.e. with similar reinforcer amounts and similar GAS and GRS sensitivities), dithering will occur later (or not at all) in trait risk prone and earlier in trait risk averse rats. In particular, an individual given an anxiolytic drug (which affects only the GIS and temporarily decreases risk aversion) will approach closer to the danger before stopping and will not show dithering behaviour.

States result in behavioural change on a timescale of milliseconds. This necessitates complex control by hierarchically organised systems/subsystems that have parallel control of acts, actions and goals (McNaughton, DeYoung & Corr, 2016). Even in terms of goals, there is dynamic control where the focus may shift between approach, avoidance and conflict extremely rapidly (Figure 1A).

This complexity means that these systems have been subject to phylogenetic change via a large number of small linked steps, each the result of a simple mutation. Each parameter of general attraction and repulsion, and each specific behaviour among elicited motivational responses (such as mating display rituals), has been, and continues to be, fine-tuned individually and, except in an ecological sense, independently of other parameters or action patterns.

Traits, in contrast, operate on a timescale linked to lifespan, population and phylogeny. They reflect sensitivities, and so simple control, of systems/subsystems. This simplicity is a computational necessity. Where different levels of a system are selected depending on, say, the level of state anxiety; a factor (e.g., endogenous benzodiazepines (BDZs) that controls trait anxiety must act on the whole system, since it will determine which current level of the state system is selected by some current input (Figure 1B).

This simplicity means that trait control of state systems has been subject to global phylogenetic change (but still as a result of a multitude of mutations, making the genetics complex). The resultant control factors are, therefore, ideal for a bottom-up approach to personality.

### 1.6 Bottom-up personality biology?

There is a strong contrast between ABCD *states*, which reflect rapidly changing activity in complex neural systems and core ABCD *trait* control, which is not only relatively stable on the timescale of the life of an individual but appears highly conserved in phylogeny. That said, states and traits reflect the impact of shared selection pressures. The important point for the rest of our argument is that we can explain *conserved* traits by simple factors that are instantiated at the biological level. This does not rule out traits that may emerge at higher levels but provides a foundation on which the latter must have evolved, developed, and on which they depend for their current expression. Critically, we should not ascribe to higher level emergence any ABCD pattern that we can explain by lower level biology.

What follows is in two parts. The first deals with the “*why*” of fundamental personality traits; to which the answer depends on the nature of evolution. The second deals with the “*how*”; to which the answer is neuroscience.

My argument will be that a conserved *trait* reflects an adaptive function (or a series of such) that links both to a surface coherence for the lexical labels we use and to a fundamental molecular-level modulator and so connects the two. That is, our fundamental biological explanations will map approximately to existing lexical surface structure because both map to a class of adaptive function with a constrained evolutionary path.

In what follows, I will also describe complex intervening *state* systems that allow traits to be expressed, via behaviour, as detailed patterns. But the details are provided simply to show that this mapping is possible, and they can be ignored for the purposes of trait arguments and lexical:neural mapping.

## 2. The argument for neural-level personality factors

In what follows, I will argue that the nature of biological explanation (and particularly the fact that such explanation can span multiple levels) means that we must look at both the why and the how of personality. Importantly, evolved systems must pass through a functional filter (where only the “fittest” survive), and evolution and development both require that the next iteration of a system must morph only marginally from the previous one in a way that constrains what would otherwise be sensible engineering solutions to problems.

### 2.1 Types of biological explanation

**Figure 2.**
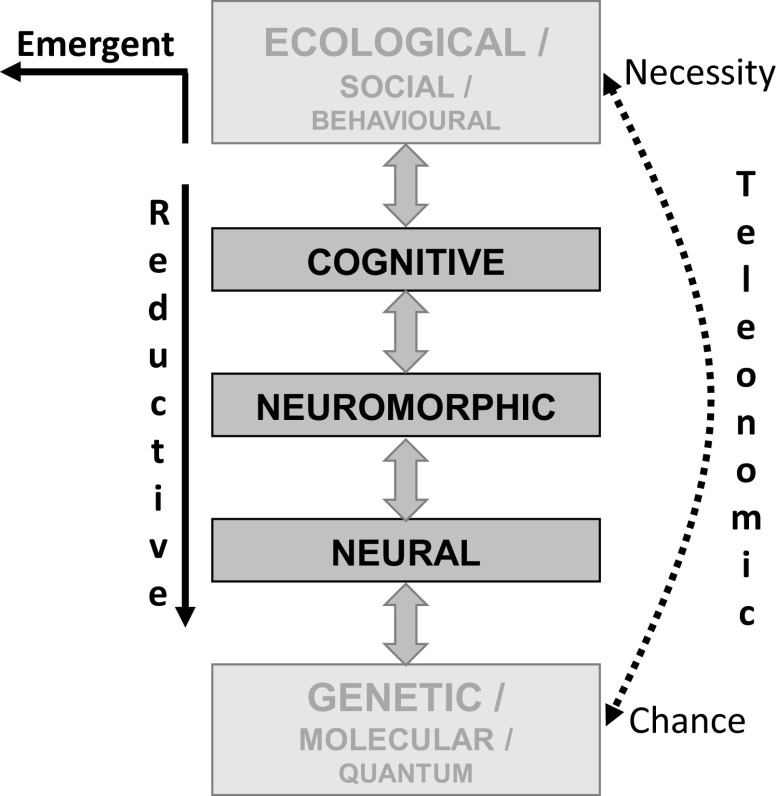
Levels of explanation for personality neuroscience. Conventional reductive explanation for phenomena at the observational (including social and ecological) levels can usefully descend through cognitive and neuromorphic levels to constructs at the neural level (McNaughton & Smillie, 2018). That is, a detailed theory expressed in neural terms can, potentially, fully explain an observation at the personality level. However, at each of these levels, emergent properties may need to be invoked, and with some aspects of behaviour, a full explanation may include emergent properties at the behavioural level. In general, social and ecological factors will operate at too high a level to provide useful detailed explanation of personality, while genetics, molecular (biochemistry and chemistry) and quantum mechanics will usually provide details that confuse more than they explain. Teleonomic (evolutionary) explanations span and link the other levels of explanation.

Since our goal is to *explain* personality, it is probably wise to first look at the different types of explanation that we could be calling for. We need to note that explanation can involve a range of levels (Figure 2) and that a low-level explanation can be entirely satisfactory (albeit complex to work through) at a very high level provided emergent properties are not involved. Once we understand its detailed impact, the details at the low level may completely explain something that appears very complex at the high level. Equally importantly, certain forms of explanation (particularly evolutionary ones) can operate across levels – providing higher order explanations of our explanations. Even with single cells, we may need ecological level explanations to understand population genetics-level changes (Black, Bourrat & Rainey, in press).

The key issue, for levels of explanation, is the level of observation at which we wish to attribute causal agency. Our attribution of cause can be emergent, reductive or teleonomic (Figure 2).

With emergence, the key feature is that the proposed causes are at the level of observation. Critically, consideration of the next level of explanation down does not fully explain the phenomenon. Also, significantly, the same explanation can be provided across a range of different systems. If you have never seen a murmuration of starlings (or other birds), you should try and make a trip to do so. This is a spectacular object, formed from myriad birds, that looks as if it is a unique life form (see, e.g., King & Sumpter, 2012, Figure 1 for a photograph). Starlings are not the only birds that display this characteristic (personality) behaviour, but the key point is that very similar high-level principles explain the form of, for example, the shoaling behaviour of fish (Herbert-Read et al., 2011, p. 18726), where “although the positions and directions of all shoal members are highly correlated, individuals only respond to their single nearest neighbour”. Critically, the explanations are ones that explain the general form of unique objects where no specific pattern will ever repeat exactly.

With reduction (often seen as the “real” form of scientific explanation), there is a succession of lower levels of potential explanation for a phenomenon. For cognitive neuroscience, the ideal cascade is from neurons to circuits to choices. Here, we can explain (actually quite simply) the individual actions of a starling within the flock, but it takes a computer (and a few assumptions) to model the resultant murmuration. The neuroscience model does not answer “why” questions at the emergent (group) level but does answer “how” questions at the level of the individual rather than the group.

With teleonomy (Pittendrigh, 1958), explanation must account for the interaction of chance and necessity (Monod, 1972) – of mutations and adaptive pressure. It can apply at any of the conventional levels of explanation and so, particularly for our current analysis, can often bind levels of reductive explanation. Teleonomy is an inherently complex *historical* adaptive path. But, provided we are cautious, we can often convert it to superficially teleological shorthand: The murmuration is “for” avoiding hawks. In the remainder of this paper, I have referred to teleonomy as “teleonomic purpose” to remind you of this functional shorthand while also flagging that teleonomy is explicitly not intentional or purposive in the teleological sense.

Because teleonomic purpose is complex, it can help, initially, to cheat with teleology. Provided there is not a zoologist close by, we can begin to understand the evolution of the eye by saying that it evolved “for” seeing. Once we get an idea of why it has its different parts from an engineer’s perspective, we can then revert to a more scientifically respectable explanation and translate our understanding back to teleonomic purpose. The full story, with diagrams, for the eye (single chamber and compound) is told by Nilsson (2013) but is, in essence, a simple progression of steps conferring ever increased survival capacity via detection of*: light presence (non-directional photoreception detecting) → light direction (directional photoreception) → pinhole image (low resolution vision) → focussed image (via lens in pinhole)*. Our single chamber eyes have further improvements such as an iris for sharper focussed images and colour for, for example, detection of fruit ripeness. Each step is of advantage, and each changes the adaptive equations in a way that sets the scene for evolution of the next step. In practice, such progressions involve crooked, constrained, paths. Critically, the lack of rational engineering of the “end product” (which is actually a current snapshot of events and expected to alter in the future) allows us to explain why a human eye has a “blind spot” but an octopus eye does not and why cavefish and mole-rats have vestigial eyes but are functionally blind.

### 2.2 An emotion is a teleonomic purpose

The most basic forms of what we can call state emotions are controlled by complex evolved brain systems. The problem being solved by such systems is that of the adaptive conflict between opposing conditions. For example, with blood clotting, a high level is maladaptive leading to embolism and so strokes, while a low level is maladaptive and results in excessive bleeding (as in haemophilia); so each is adaptive under different conditions. Emotional systems solve this problem by adjusting systems to the current situation.

In the presence of threat, blood clotting will be increased (reducing blood loss after injury); whereas, in safety, clotting will be decreased (reducing the risk of strokes). Over evolutionary time, the constant adaptive challenges resulting from threat, coupled with many mutations, have created a coincident suite of reactions. These reactions are not all ones we are aware of and include (Cannon, 1936) increased: blood sugar (for muscular energy); adrenaline (to reduce fatigue); vasodilation (to maximise muscular exertion); red blood corpuscles (in anticipation of bleeding); respiratory function (to support great effort) and blood coagulation (to reduce blood loss).

In general, recurring necessity has produced correlated ABCD changes for which their teleonomic purpose is the simplest referent for the emotion name (e.g., “fear”) across species. On this view an emotion can be defined as a *set of ABCDs* that share a teleonomic purpose (McNaughton, 1989) or more loosely, all those reactions that have evolved “for” some specific “function” (Table 3).

**Table 3.** Emotions defined via teleonomic purpose and “adaptive value”. Note that, for example, we would expect love to be strong in albatrosses as a chick needs food supplied by both parents if it is to survive

Emotion	Teleonomic purpose/goal/function	Adaptive value	
Lust	Copulation	Offspring	
Fear	Repulsion from danger	Survive threat	[ → offspring]
Anxiety	Cautious approach to danger	Get food despite threat	[→ offspring]
Love, etc.	Pair bonding	Offspring survive through care	

Not only does the “necessity” component of such basic emotions stay fairly constant but, once evolved, the lower level neural subsystems are hard to change without catastrophic consequences and so mutations tend to add new control systems on top of the old, Thus, teleonomic purpose tends to conserve state and trait control and to build hierarchical systems (from both a neural and a behavioural perspective).

### 2.3 Teleonomic purpose and state control

**Figure 3.**
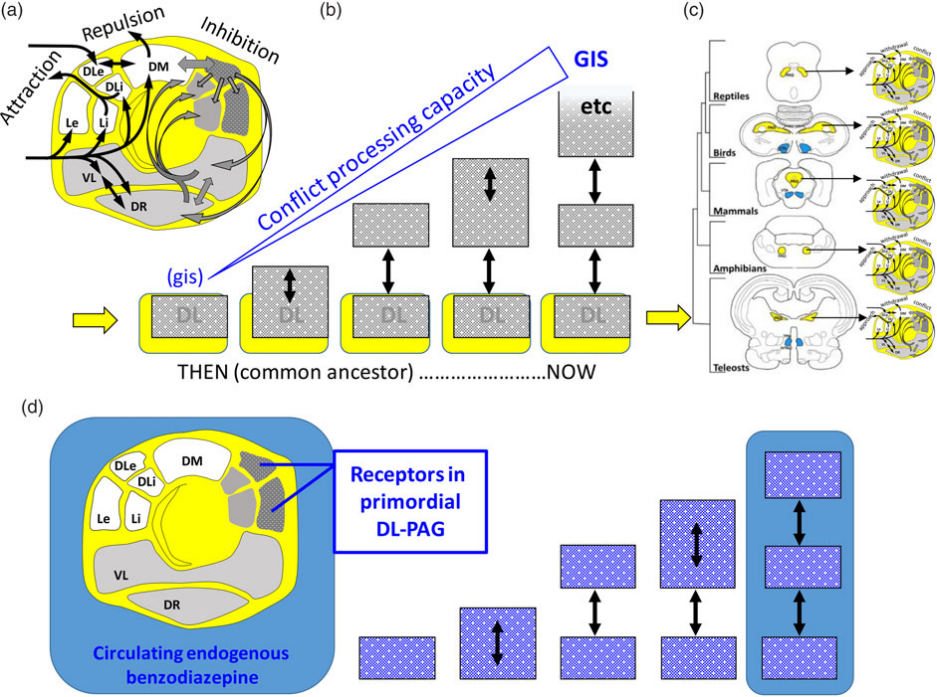
Conservation of the primordial PAG with expansion of motivational controls systems such as the GIS. (a) Attraction, repulsion and inhibition (conflict) modules within the PAG. (b) This core is retained during phylogenetic expansion as higher levels of control are added. (c) Location and appearance vary across phyla but fundamental organisation is not changed. (d) During expansion, receptors for endogenous benzodiazepines are conserved in those structures for which benzodiazepine modulation is not maladaptive. Panels (a) and (c) based on Silva and McNaughton (2019), with permission. DM: dorsomedial PAG; DL: dorsolateral PAG; L: lateral PAG; VL: ventrolateral PAG; DR: dorsal raphe nucleus; e: external part; i: internal part.

The lower level conservation of state system control is particularly clear with the motivational systems that process goals. RST (see Corr, 2008 for overview) is based on three distinct primary functional systems (Gray & McNaughton, 2000; McNaughton & Corr, 2004) that control approach to a positive goal (GAS), withdrawal from a negative goal (GRS) and inhibition of behaviour linked to both attraction and repulsion with production of other behaviour (e.g., risk assessment behaviour) when there is goal conflict (GIS). Recent analysis of subcortical control of these systems (Silva & McNaughton, 2019) shows that all three are present within the PAG, the lowest level of the brain at which integrated goal control exists (Figure 3A).

The organisation of the PAG, in relation to the three RST systems, appears to have been conserved across vertebrate species (Figure 3C). The implication of this is that in a common ancestor PAG can be viewed as the primordial origin of the RST systems. If we focus on the GIS (which is defined by anxiolytic drug action and so can be identified via relevant receptors), we can then see a progression (Figure 3B, D) from a primitive module in the homolog of the current dorsolateral PAG, through its expansion to allow more extensive conflict processing, to a split into a lower and a higher level module, and then similar progressive splitting into distinct ever-higher-level modules. The resultant progressive rostral phylogenetic expansion generates a *control hierarchy*, the modules of which have a common teleonomic purpose/global function and where the lower levels conserve “quick and dirty” methods as the higher levels develop ever more “slow and sophisticated” ones (LeDoux, 1994). This GIS controls goal-conflict processing in state terms, which approximates to control of state “anxiety” (Gray, 1977, 1982; Gray & McNaughton, 2000).

### 2.4 Teleonomic purpose and trait control

In relation to anxiolytic action, BDZ receptors appear to have been particularly conserved during evolutionary expansion of the brain. They occur first in higher order bony fish (Nielsen, Braestrup & Squires, 1978) and are retained in all phylogenetically later vertebrates with progressive changes in their subunit structure (Hebebrand, Friedl, Breidenbach & Propping, 1987). Their effects, which as I noted *define* the GIS, are fundamentally the same in vertebrates from fish to humans on a wide range of behaviours. The endogenous BDZ receptor ligands appear to be systemically circulating neurohormones, as do a range of other compounds that allow us to create neurobehavioral models of human psychiatric disorders in fish (see Soares, Gerlai & Maximino, 2018). In neuromodulators and hormones in general, and BDZs in particular, we have compounds that control the sensitivity of entire conserved hierarchical systems. It seems reasonable, therefore, to see the endogenous BDZ receptor ligands, modulating GIS activity, as contributing to a specific form of trait control of anxiety (Lehmann, Weizman, Leschiner, Feldon & Gavish, 2002).

### 2.5 Complex circuits but simple modulation

**Figure 4.**
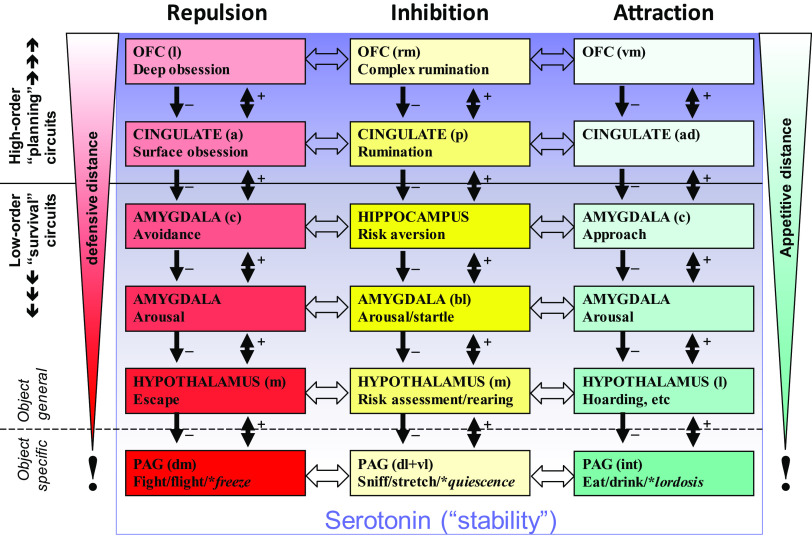
The relationship of modulators to hierarchical systems. Goal attraction (GAS), goal repulsion (GRS) and goal inhibition (GIS, activated by conflict between goals) are each controlled by systems in which modules are organised hierarchically in relation to motivational distance (contacting-distant) and neural location (caudal-rostral). Conservation of modulatory control during phylogeny (Figure 3) means that hormonal compounds, for example, BDZ receptor ligands, and neuromodulators, for example, serotonin, can target all the modules of a specific system (as with BDZ and the GIS) or all the modules of several systems (as with serotonin). Note that in the case of serotonin, its effects (indicated by the gradation of the purple shading) appear to be to shift control from lower to higher levels of the systems (Carver, Johnson & Joormann, 2008) rather than to increase or decrease activity across an entire system. Figure based on Silva and McNaughton (2019). *Static postures that allow avoidance, conflict resolution or approach, respectively.

An important consequence of hierarchical expansion of modular systems, when this is coupled with conservation of the particular types of hormonal modulatory receptors, is that the ligands of those receptors can target whole systems/subsystems. This is also true of neural modulatory systems, such as the monoamines, where their collateral branches will retain diffuse connections when such systems increase in complexity. So, while these neurally very complex hierarchical functional systems are capable of sophisticated state reactions, their conserved functional role and conserved modulatory control mean that their trait control is intrinsically simple; I would argue that we can identify them with the relevant modulators (Figure 4). I will give just one example of each type of system here, but (as we will see in the next section) there will be many examples of each type.

*Endogenous* BDZ *receptor ligands (yellow in* Figure 4 *)*, as we have noted, act on (and indeed define) the GIS. We can, therefore, take variation in the blood levels of the ligands (which include both agonists and inverse agonists) as altering trait-GIS and so a form of trait “anxiety”. Variation in receptor density (in structures or in key afferent systems, shown by variation in colour density in the figure) can fine-tune the extent of control. (The same will be true with variation in terminal density for neuromodulators.)

*Monoamine neuromodulatory transmitters* (noradrenaline, dopamine and serotonin – shown as purple in the figure) act on the GAS and GIS and GRS. They appear to bias (Carver, Johnson & Joormann, 2008) at what level of the system control is exerted (illustrated by the gradation of colour in the figure). This motivation-system-wide control is consistent with the idea “that the serotonergic and dopaminergic systems are the major biological substrates of Stability and Plasticity, respectively” (DeYoung, 2015, p. 47).

An important point is that these two types of modulatory control (hormone-like and diffuse neural innervation) can overlap in terms of their targets. It follows that their trait-level effects on patterns of behaviour will interact in some cases.

### 2.6 Independent factors but oblique aetiology

At this point, it will be useful to consider a mirror to the view that psychopathology results from extremes of traits and ask whether therapeutic drugs can inform us about the traits whose extremes they are reducing. Interestingly, the effects of a range of clinically effective drugs suggest that neural modulators can act *as independent factors* at any level of the “personality hierarchy” (see DeYoung, 2015). Specific serotonin reuptake inhibitors, changing the levels of serotonin quite generally in the brain (e.g., Figure 4), would act at the metatrait level if, as suggested by DeYoung, they alter stability. Ketamine has been shown to be effective in all the “neurotic” disorders (Andrews, Stewart, Morris-Yates, Holt & Henderson, 1990; World Health Organization, 2010) including cases that are resistant to other pharmacological treatments of generalised anxiety, social anxiety, panic, obsession and depression (Feder et al., 2014; Glue et al., 2017; Loo et al., 2016; Rodriguez et al., 2013; Zarate et al., 2006; Zhang & Hashimoto, 2018). This suggests there is a neural modulator that is sensitive to ketamine and that alters neuroticism and so operates at the domain level. Panic (mediated via the PAG; Silva & McNaughton, 2019) could also result from high sensitivity of lower levels of the repulsion system (Figure 4, graded red colour), potentially mediated via CCK4 (Benkelfat et al., 1995; Wang, Valdes, Noyes, Zoega & Crowe, 1998) and operating at the aspect level. Similarly, anxiolytics, in the sense of drugs that affect generalised anxiety but not panic, phobia, obsession, etc (McNaughton, 2018), and particularly ligands of the BDZ receptor, can be seen as operating on “trait anxiety” at the facet level, with the related endocannabinoids potentially controlling stress, anxiety and fear (Morena et al., 2019).

A key feature for each of these underlying biologically based personality factors is that their teleonomic purpose can be mapped to emotion labels. Thus, each modulator can be at least approximately identified with a lexically simple label (stability / neuroticism / repulsion / anxiety) and, when extreme, with a form of psychopathology or a risk factor for such psychopathology.

This biology directly impacts what we may want to say about personality at the lexical level. These biological entities (serotonin systems, BDZ receptors) are fundamentally independent as trait-control variables but are not independent in how these traits are expressed in surface ABCD (including language): they have shared developmental trajectories (e.g., there can be parallel impacts on a range of key systems from traumatic events); critically, there are interactions between state systems (the presence of a high level of state attraction will subtract from the expression of a particular level of state repulsion). As a result, ABCD effects will be superficially oblique at the level of surface behaviour, and so lexically, even when the underlying trait sensitivities are functionally orthogonal. A related problem is determining the true source of morbidity when high panic sensitivity may condition anxious behaviour despite a normal anxiety system, high anxiety sensitivity may elicit panic attacks despite a normal panic system and when both systems may be excessively sensitive generating comorbidity (McNaughton & Corr, 2016).

At least for conserved functional systems (arguably all those involved in psychiatrically problematic emotions), then, predictive explanation must start with neural-level traits and then determine ABCD patterns. We must be prepared for cases where, for example, the trait of “tameness” in foxes has a suite of linked behavioural characters packaged with dog-like physiognomic ones (Trut, 1999), and, less obviously, where two sets of behavioural characters that one might expect to be distinct for other reasons are packaged together as expressions of a single trait because they share a single biological control factor.

### 2.7 Mapping personality to the brain

From this perspective, many fundamental outputs of “personality” can be viewed as effects of long-term global factors that control the sensitivities of hierarchical modular systems (Figure 4). These global factors will usually be endogenous compounds (acting in a hormonal or neuro-hormonal fashion) such as serotonin (controlling “stability”), endogenous BDZ ligands (controlling “trait anxiety”) and perhaps cholecystokinin (CCK) acting within the PAG (controlling “trait panic”). These traits, when at extreme high or low values, will make a key contribution to psychiatric disorders (which could be termed, e.g., “anxiety” disorder and “panic” disorder) or risk factors (e.g., neuroticism). However, such traits (as patterns of explained ABCDs) may not match your current usage of terms like “anxiety” since different people use them with quite different meanings (McNaughton, 2018).

Specific modulatory systems will control a background sensitivity that controls the production of *coherent* patterns of bodily and behavioural activity. However, these trait sensitivities act on systems that generate specific *adaptive* behaviours that are highly dynamic. Changing events, and the organism’s own actions, will mean that control of the current state passes rapidly (on a millisecond scale) both between systems and among modules within the hierarchy of each system.

To say that trait control is static in comparison to the rapid interplay between systems’ states is not to say it is fixed and especially does not imply any strong genetic control. The heritability of neuroticism and anxiety are both about one-third that of generalised epilepsy (Anttila et al., 2018). Like generalised epilepsy, they have multiple (potentially overlapping) genetic factors that can contribute to the adult phenotype (Genetics of Personality Consortium, 2015; Luciano et al., 2017; Nagel et al., 2018), and these combine with a range of environmental factors in development to determine the nature of the adult system. An instructive comparison, here, is between attention-deficit hyperactivity disorder (ADHD) and phenylketonuria (PKU). ADHD has about a 70% heritability (Cortese, Faraone & Sergeant, 2011; Durston, 2010) but is highly polygenetic with small dose-like contributions from many genes (Faraone & Khan, 2006; Neale et al., 2010). PKU occurs when a mutation of the phenylalanine hydroxylase gene is inherited from both parents, blocking the synthesis of phenylalanine hydroxylase, and producing a damaging build-up of phenylpyruvic acid, which can largely be prevented by placing cases on a low phenylalanine diet. As reviewed by Stevenson and McNaughton (2013, p. 63),

The biochemical and neural pathologies of PKU and ADHD are quite distinct in their causes and detail; but they result in the disorder in the brain of large amino acid levels, dopamine and white matter that are very similar and could explain the overlap of symptoms within and between the PKU and ADHD spectra. The common deficits affect visual function, motor function, attention, working memory, planning, and inhibition.

Here, we have two phenotypes with radically different genetic causes and quite different paths to dysfunction but a subset of very similar neural system abnormalities that map to a subset of shared ABCD pattern abnormalities. The key to understanding the ABCD patterns (involving vision, motor control, attention, working memory, planning and behavioural inhibition) and their variations between the disorders is to map them to variation in dopamine systems and to distinct rostral and caudal elements of the GIS (Stevenson & McNaughton, 2013, Figure 5, p. 78) and perhaps also to overall GIS sensitivity for the inattentive ADHD subtype (Sadeghi et al., 2018).

Finally, to say that some key fundamental motivational traits are best explained via large-scale neural system modulation is not to identify all traits each with a single such modulator or all disorders with a single trait. Some coordinated, evolutionarily old, responses (such as the stress response) can involve the concerted activity of several modulators (cortisol/corticosterone, serotonin, noradrenaline); others may involve the summated separate actions of more than one such modulator (with panic-proneness likely controlled by both more general serotonergic “stability” and a CCK-sensitive panic-specific trait) and specific disorders may involve the combination of high levels of more than one trait. For example, the general class of neurotic disorders appears to require an extreme of both neuroticism and an extreme of trait panic or trait anxiety, or trait obsession (McNaughton & Glue, 2020). Other traits (like murmuration) may be emergent. Also, as suggested by a referee, there may be “phylogenetically newer traits, which tend to be more specialized and which are relevant to fewer contexts (e.g., rejection sensitivity, gregariousness)”. However, before invoking complex, especially non-biological, explanations for such traits, I think it will be important to be sure that existing simple biological factors cannot account for the large-scale patterns of ABCD (as in the case of PKU discussed in the previous paragraph).

## 3. Conclusions

In summary, at least for motivation-related traits, I think we should move from linguistic description to neuroscientific explanation. Within a linguistic description, a personality factor equates roughly with some lexical definition, with the danger that a scale may reflect no more than the presence of a set of synonyms (Cooper, 2019). Where a lexically derived trait is a genuine label for a superficial ABCD pattern, it assumes coherence of the items involved, but it predicts weakly by extrapolation or emergence. In this context, we can view the tendency to form a murmuration as a starling trait and the form of any particular murmuration as a specific ABCD case that conforms to the general pattern. Within a causal neuroscientific explanation, a simple personality factor equates roughly with the strength of a modulator. It is a system sensitivity and (with a sufficiently detailed description of the system) predicts ABCD patterns strongly via teleonomic purpose and reduction. Importantly, the use of deep explanations by neuroscience means that a neuroscientific trait theory will predict both coherence and occasional *apparent* incoherence in ABCD variation. For example, our neuroscientific understanding of the evolution of the eye allows us to account for the presence of a blind spot in one phylogenetic lineage (which includes us) and not another (which includes the octopus).

My key argument has 6 elements (with anxiolytics and trait-GIS as an example):

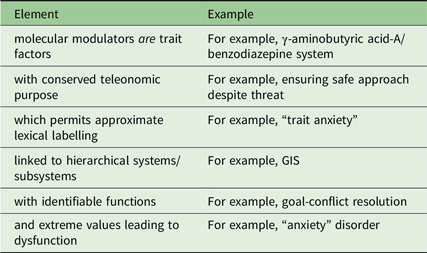



With sufficient system-level information (e.g., Figure 4), this means that we will be able to predict ABCD state and trait changes. It also follows that, where they are ligands of the relevant receptors, drugs can be used to develop biomarkers for state (and so by derivation trait) measures of these systems. In the case of trait GIS (the basis for one possible “trait anxiety” factor), we already have at least one such biomarker (McNaughton, 2018; Shadli et al., 2019; Shadli et al., 2015) with others awaiting drug validation (Lockhart, Moore, Bard & Stafford, 2019; Moore, Gale, Morris & Forrester, 2008; Moore, Mills, Marsham & Corr, 2012).

Biomarkers for the trait control of systems can then be used to anchor and, in many cases, define scales via the underlying biological cause of ABCD consistencies. This is essentially the opposite of current supposed-RST scales that have been derived lexically and not even validated against the key biological constructs of RST. This will force us to redefine lexical terms as required. For both state and trait, “anxiety” (as defined by anxiolytic drugs) is *not* panic, fear, obsession or depression. For trait anxiety, it will also allow us to redefine or rename current scales determining the neural relationships (if any) between Taylor’s manifest anxiety (Taylor, 1953, 1956); Spielberger’s trait anxiety (Spielberger, Gorusch, Lushene, Vagg & Jacobs, 1983); BIS/BAS (Carver & White, 1994) and personality inventory for DSM-5 anxiousness (Markon, Quilty, Bagby & Krueger, 2013).

So, should we start with biology and look for neural-level factors? I would argue that, *with the fundamental motivational systems* embedded in RST (Figure 4), it is working for me. Given the assumption that there are many such conserved fundamental systems, it should also work more generally. This neural-level approach will clearly not explain all personality traits, but it will provide a solid foundation on which to build understanding of traits that emerge at higher levels.

## Footnotes

1 Drawn to my attention by Luke Smillie.

2 HEAXACO is a 6 rather than 5 primary factor model, where the 6 factors are honesty-humility (H), emotionality (E), extraversion (X), agreeableness (A), conscientiousness (C), and openness to experience (O).
